# Incomplete concordance between laboratory and pathologic findings on post-induction kidney biopsy in pediatric patients with proliferative lupus nephritis

**DOI:** 10.1007/s00467-025-06736-y

**Published:** 2025-03-25

**Authors:** Robin Raschke, Clarkson Crane, Robert Sheets, Noureddin Nourbakhsh, Nadine Benador, Elizabeth Ingulli, Katayoon Shayan, Peter Yorgin, Caitlin Carter

**Affiliations:** San Diego Department of Pediatrics, Rady Children’s Hospital, San Diego and University of California, La Jolla, CA USA

**Keywords:** Children, Proliferative lupus nephritis, Biopsy, Outcomes, Kidney failure

## Abstract

**Background:**

Proliferative lupus nephritis (LN) is associated with increased risk of progression to kidney failure. After initial kidney biopsy, the utility and timing of subsequent biopsies is unknown. There is known discordance between the laboratory parameters used to diagnose LN and the histopathologic classification. We explore the utility of a subsequent kidney biopsy in guiding treatment of LN to determine the factors that warrant follow-up kidney biopsy.

**Methods:**

We conducted a single center retrospective cohort study of 30 SLE patients who underwent serial kidney biopsy for LN. Subjects were stratified based on their Childhood Arthritis and Rheumatology Research Alliance (CARRA) renal response into complete renal response (CRR) and incomplete renal response (IRR) groups at the time of second biopsy.

**Results:**

Among 30 patients with LN, 11/18 in CRR group and 11/12 in IRR group had persistent proliferative nephritis at 1 ± 0.3 years after initial biopsy. Only SLEDAI score was associated with an increased risk of persistent proliferative nephritis (*p* = 0.03). Initial CARRA response category was associated with outcome at last follow-up (mean 4.5 years), with 11/18 CRR and 3/12 IRR achieving CRR at last follow-up at mean 4.5 years (*p* < 0.001). Kidney biopsy directly impacted clinical decision in 7/18 CRR patients in the CRR group who had therapy escalated or reduction withheld due to biopsy findings.

**Conclusions:**

Available laboratory markers in LN are insufficient to identify children with ongoing proliferative nephritis. Follow-up kidney biopsy may be warranted for children with CRR at 1 year after initial biopsy.

**Graphical abstract:**

**Figure:**
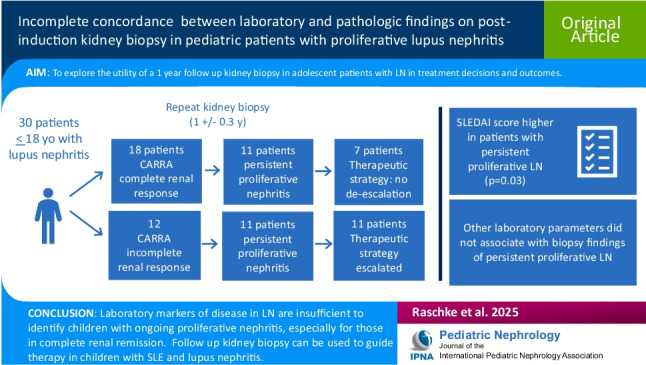
A higher resolution version of the Graphical abstract is available as Supplementary information

**Supplementary Information:**

The online version contains supplementary material available at 10.1007/s00467-025-06736-y.

## Introduction

Systemic lupus erythematosus (SLE) diagnosed during childhood is associated with higher rates of mortality [1] and lupus nephritis [2] compared to adult-onset SLE. Lupus nephritis (LN) itself is independently associated with higher mortality and proliferative lupus nephritis (ISN/RPS class III and class IV) is associated with an increased risk of progression to kidney failure [1]. In current practice, the diagnosis of active lupus nephritis is suspected when a patient has clinical findings including proteinuria, hematuria, hypertension, or a decline in estimated glomerular filtration rate (eGFR) and is confirmed with kidney biopsy [3]. Once a diagnosis of LN has been confirmed on initial kidney biopsy, the utility and optimal timing for subsequent biopsies in LN has not been established. Some programs have concluded that a subsequent kidney biopsy should be performed only in the setting of worsening clinical or laboratory evidence of disease, while others schedule protocol kidney biopsies at various time intervals, including following induction therapy, or when the patient is receiving maintenance immunosuppression [4].

There is known discordance between the laboratory parameters used to diagnose LN and the histopathologic classification. Silent LN has been described in patients without laboratory manifestations of nephritis but who have biopsy findings consistent with histologically active glomerular disease [5–7]. In addition, active nephritis may be suspected in patients with proteinuria or decline in eGFR who are found to have glomerular and interstitial scarring in the absence of active inflammatory glomerular disease. Often clinicians are uncertain about the impact of titration in immunosuppression when relying solely on laboratory parameters.

Clinical response rates are known to be less than 50% after initial therapy and rates of complete histologic response are reported to be even lower, approximately 20% [3]. Our practice has been to obtain surveillance kidney biopsy in patients with proliferative LN at approximately 1 year after the initial kidney biopsy. In this retrospective analysis, we describe the histopathologic findings of LN activity on second kidney biopsy in children diagnosed with LN to explore the utility of a subsequent kidney biopsy in guiding treatment of LN after induction therapy. In addition, we compare patients with and without complete renal remission, to determine if the standard laboratory signs of kidney disease can be used to target repeat kidney biopsy to the patients most likely to benefit from the information obtained.

## Methods

### Subject identification

We performed a single-center retrospective cohort study of all patients with SLE who underwent kidney biopsy for LN between January 1, 2011 and December 31, 2022 at Rady Children’s Hospital, San Diego (RCHSD). Subjects were eligible for inclusion if they had had two kidney biopsies during the study period. Inclusion criteria were age ≤ 18 years at the time of first biopsy, diagnosis of proliferative lupus nephritis (ISN/RPS class III or class IV) on either the first or second biopsy, and a pathology report from both biopsies that included LN class, and NIH activity and chronicity scores. Patients either received (1) a standard of care biopsy at 1 ± 0.3 years after their first biopsy or (2) a biopsy based on clinical concern. Exclusion criteria were insufficient laboratory data to assess lupus or kidney activity (serum creatinine, complements, urine protein to creatinine ratio (uPCR)) or insufficient histologic data to classify by ISN/RPS classification. Patients with a first kidney biopsy prior to the study or first kidney biopsy at another institution were excluded. Patients were identified using the electronic health record (EHR) and charts were manually reviewed for inclusion and data extraction.

### Demographic and laboratory characteristics

Demographic and laboratory data were obtained for all subjects from the EHR and compiled in a Microsoft Excel database. Sex, race, and language were self-reported. Laboratory testing was performed at the laboratory of the patient’s choosing. Systemic lupus erythematosus disease activity index (SLEDAI) scores were calculated at the time of kidney biopsy as indicators of global lupus activity [8]. Estimated glomerular filtration rate (eGFR) was calculated based on serum creatinine using the CKiD U25 equation [9]. Proteinuria was monitored using random urine total protein/urine creatinine ratios, reported in g/g. Hematuria was monitored by urinalysis and urine microscopy which was reported in red blood cells per uL.

### Biopsy

Consistent with our clinical practice, the majority (58/60) of the included kidney biopsies were performed by an attending pediatric nephrologist or by a pediatric nephrology fellow with attending supervision. Real-time ultrasound guidance by an ultrasound technician was used for biopsies performed by the nephrology team. The remaining two biopsies were performed by interventional radiology at RCHSD, one using computed tomography guidance and one using ultrasound guidance. All biopsies were performed under general anesthesia. Pathology analysis was performed by pediatric pathologists at our institution. ISN/RPS classification and NIH activity and chronicity scores were reported [10]. The decision regarding timing of all kidney biopsies was made by the treating nephrologist(s) in collaboration with the treating rheumatologist(s). Biopsy complications were defined as any admission to the hospital after biopsy for pain, bleeding, infection, or requirement for blood transfusion or other intervention. Asymptomatic hematomas that did not require intervention were not included due to lack of consistent follow-up ultrasound.

### Comparison by CARRA response

Subjects were stratified based on their Childhood Arthritis and Rheumatology Research Alliance (CARRA) renal response into complete renal response (CRR) and incomplete renal response (IRR) groups at the time of the second biopsy, with IRR group comprising patients with a CARRA partial response and CARRA failure [11]. The groups were compared for demographic, laboratory and pathologic findings at the time of each biopsy, and induction treatment.

### Pathologic classification

Subjects were classified as having improved, worsened, or unchanged proliferative nephritis based on the results of the second kidney biopsy. Subjects who developed new class III or class IV proliferative nephritis were considered to have worsened disease. Subjects who had persistent class III or class IV disease were considered to have persistent proliferative nephritis. Subjects who had class III or IV nephritis that remitted to class II or lower were classified as having improved disease. Subjects with class V disease in addition to proliferative (class III or IV) disease were classified according to their proliferative class.

### Follow-up

Decisions regarding changes to treatment were made by the treating nephrologist and rheumatologist. Increase in dosage or addition of a new medication were considered escalation in therapy, while cessation or dose reduction were considered de-escalation in therapy.

Patient response was evaluated for clinical remission status up to most recent data after the second biopsy to assess for change in CARRA response.

The study was approved by University of California, San Diego IRB (200870X). The datasets generated during and/or analyzed during the current study are available from the corresponding author on reasonable request.

### Statistical analyses

Demographic data were not distributed normally; therefore, a non-parametric Wilcoxon paired *t*-test was used for comparison of continuous variables. The chi-squared test was used for categorical data between remission groups. Single predictor and multiple predictor logistic regression models were utilized to assess the relationship between laboratory parameters and histologic findings on second biopsy. Math Cracker software was used for statistical calculations. Regression analysis was done using RStudio software. Differences were considered statistically significant if *p* < 0.05.

## Results

### Study population

There were 135 patients with SLE and nephritis identified in the EHR. Of these, 63 (47%) patients had at least two kidney biopsies between 2012 and 2022, and of those 30 (48%) subjects met criteria for inclusion in the analysis. The primary reasons for exclusion were incomplete clinical or biopsy data, no diagnosis of proliferative LN, or follow-up biopsy outside of the designated time.

Of the 30 patients who met inclusion criteria for analysis, 18 had achieved CRR and 12 had incomplete or no response (IRR) after initial therapy. All patients in the CRR and six of the 12 patients in the IRR had second biopsy performed as protocol biopsy. The remaining six patients in the IRR group had the second kidney biopsy performed for persistent or worsening proteinuria (4/12) hematuria and weight loss (1/12), or joint pain and hypocomplementemia (1/12).

The median age at time of the first biopsy was 14.0 years (IQR 12.6–15.9). The cohort was majority female (28/30) and the majority identified as LatinX (21/30). There were no significant differences in age, sex, or self-identified race between the CRR and IRR groups (Table 1).

**Table 1:** Demographic, clinical, and pathologic findings at time of initial kidney biopsy with induction therapy regimen for lupus nephritis among CARRA complete and incomplete response groups

	All patients (*n* = 30)	CARRA response: complete (*n* = 18)	CARRA response: incomplete (*n* = 12)	*p*-value
Demographics				
Female (*n*, %)	28 (93%)	17 (94%)	11 (92%)	
Age, years (median, IQR)	14.0 (12.6–15.9)	14.2 (12.7–15.8)	13.6 (12.5–16.9)	*p* = 0.83
*Ethnicity*				
LatinX	21 (70%)	12 (67%)	9 (75%)	*P* = 0.63
Other	9 (30%)	6 (33%)	3 (25%)	
Clinical characteristics (median, IQR)				
eGFR (mL/min/1.73 m^2^)	106.5 (62.6–123.1)	110.0 (77.6–126.7)	90.3 (53.1–121.5)	*p* = 0.45
Urine protein/creatinine (g/g)	1.20 (0.53–5.62)	0.91 (0.25–3.44)	2.20 (0.97–9.18)	*p* = 0.13
SLEDAI score	16.5 (12–22)	16.0 (11.8–22.0)	21.0 (15.3–26.5)	*p* = 0.13
dsDNA (Iu/mL)	607.5 (76.4–2004)	551.0 (62.6–2004)	720.5 (94.5–1993.0)	*p* = 1.00
C3 (mg/dL)	41 (36.8–69.8)	40 (36–63)	43.5 (32.3–85.3)	*p* = 0.58
C4 (mg/dL)	8 (7.0–10.5)	8.0 (7–10)	8.5 (4.3–13.5)	*p* = 0.93
Biopsy characteristics				
*ISN/RPS class (n)*				*P* = 0.77
Class 0–1	0	0	0	
Class 2	0	0	0	
Class 3 or 3 + 5	2	1	1	
Class 4 or 4 + 5	28	17	11	
Class 5	0	0	0	
*NIH activity score (median, IQR)*	8.0 (5.0–11.0)	8.5 (4.8–10.3)	8.0 (5.0–14.0)	*p* = 1.00
*NIH chronicity score (median, IQR)*	1.0 (0–2.0)	1.0 (0–2.3)	2.0 (1.0–2.0)	*p* = 0.84
*Crescents present on biopsy*				*P* = 0.27
Yes (*n*, %)	11 (36.7%)	8 (44.4%)	3 (25%)	
No (*n*, %)	19 (63.3%)	10 (55.6%)	9 (75%)	
Induction treatment				*p* = 0.05
Cyclophosphamide (*n*, %)	25 (83.3%)	17 (94.4%)	8 (67.7%)	
MMF (*n*, %)	5 (16.7%)	1 (5.6%)	4 (33.3%)	

### Baseline laboratory characteristics

The median eGFR among participants was 106.5 mL/min/1.73 m^2^ (IQR 62.6–123.1) and the median uPCR was 1.20 g/g (IQR 0.53–5.62). The median SLEDAI score was 16.5 (IQR 12.0–22.0), indicating severe lupus activity at the time of initial biopsy [12]. There were no significant differences in baseline laboratory parameters that reflect kidney disease between complete and incomplete responders at the time of initial biopsy (Table 1). One patient required dialysis at the time of initial presentation with LN, but recovered renal function prior to the time of the second biopsy.

### Baseline biopsy findings

The distribution of pathologic diagnosis by ISN/RPS class on first biopsy was similar between the complete response and incomplete response groups. All subjects had proliferative nephritis on initial biopsy. For this study, class III and class III + V were grouped together as were class IV and class IV + V. The most common ISN/RPS pathologic class initial biopsy was class IV which was present in 28/30 of subjects (93%). One subject in each remission group had class III LN, which represented 2/30 or 7% of the overall cohort.

Activity scores and chronicity scores were similar between groups at the time of the first biopsy. The median activity score in the CRR group was 8.5 (IQR 4.8–10.3) and the median activity score in the IRR group was 8.0 (IQR 5.0–14.0). The median chronicity score in the CRR group was 1.0 (IQR 0–2.3) and the median chronicity score for the IRR cohort was 2.0 (IQR 1.0–2.0) (Table 1). Immunofluorescent staining results were similar for both groups (Supplemental Table 1).

### Induction therapy

All subjects received pulse of three doses of intravenous methylprednisolone (30 mg/kg/dose up to maximum dose 1000 mg) and oral corticosteroids (starting dose of 2 mg/kg/day up to maximum dose 60 mg daily) as part of their induction therapy. Oral corticosteroids were tapered at the discretion of the treating nephrologist and rheumatologist to a dose of 5–10 mg/day.

Intravenous cyclophosphamide was used for induction therapy in 25/30 subjects (83%) and mycophenolate mofetil was used for induction therapy in 5/30 (17%) of subjects (Table 1). Of the 25 subjects who received cyclophosphamide, 18 received dosing per the NIH protocol and seven received lower dosing according to the Eurolupus protocol [13]. Of those who received cyclophosphamide, 7/17 in the CRR group were treated according to the Eurolupus protocol. The remainder (10/17) of patients in the CRR group and all (8/8) patients in the IRR group were treated with the NIH protocol. A total cumulative dose of cyclophosphamide ranged from 1.25 to 6.37 g/m^2^ in the NIH protocol group, as not all these patients completed a full course of treatment.

Induction doses of mycophenolate mofetil ranged from 931 to 1840 mg/m^2^/day. The median dose of mycophenolate mofetil when used as primary induction therapy was 1079 mg/m^2^/day (IQR 1000–1760). The maximum dose was 1500 mg mycophenolate mofetil twice daily.

### Laboratory findings at time of second biopsy

At the time of second biopsy, the CRR group had lower serum creatinine, higher eGFR, and lower uPCR than the IRR group. Median serum creatinine of the CRR group was 0.60 mg/dL (IQR 0.51–0.63) with a median eGFR of 103.2 mL/min/1.73 m^2^ (IQR 95.0–112.1), while median serum creatinine of the IRR group was 0.70 mg/dL (IQR 0.61–0.80) with a median eGFR of 85.1 mL/min/1.73 m^2^ (IQR 78.2–99.0). The CRR group had a median uPCR of 0.1 g/g (IQR 0.07–0.16) at time of the second biopsy, while the IRR group had persistently elevated median uPCR of 0.99 g/g (IQR 0.46–3.85) (*p* < 0.01) (Table 2).

**Table 2:** Clinical and pathologic findings at time of second kidney biopsy for lupus nephritis among CARRA complete and incomplete response groups

	All patients	CARRA response: complete (*n* = 18)	CARRA response: incomplete (*n* = 12)	*P*-value
Clinical characteristics (median, IQR)				
Age (years)	15.1 (13.7–17.3)	15.3 (13.8–17.0)	14.7 (13.6–17.8)	*p* = 1.00
Time since first biopsy (years)	1.1 (1.0–1.2)	1.1 (1.0–1.2)	1.1 (1.0–1.5)	*P* = 0.48
eGFR (mL/min/1.73 m^2^)	98.8 (84.6–108.9)	103.2 (95.0–112.1)	85.1 (78.2–99.0)	*p* = 0.30
Urine protein/creatinine (g/g)	0.20 (0.09–0.95)	0.10 (0.07–0.16)	0.99 (0.46–3.85)	***p* < 0.01**
SLEDAI score	4.0 (0.8–8.0)	2.0 (0–4.0)	8.0 (6.0–12.0)	***p* < 0.01**
dsDNA (IU/mL)	20.0 (7.0–121.5)	12.3 (7.0–121.5)	23.5 (5.3–150.3)	*p* = 0.49
Serum C3 (mg/dL)	97.5 (80.5–109.8)	99.5 (89.8–119.8)	90.0 (79.0–105.0)	*p* = 0.23
Serum C4 (mg/dL)	17.0 (12.3–22.0)	17.5 (12.3–21.3)	16.7 (11.3–26.5)	*p* = 0.97
Prednisone dose (mg/day)	5 (4.75–8.13)	5 (2.5–5)	8.75 (5–33.75)	***p* = 0.01**
Biopsy characteristics				
*ISN/RPS class (n)*				*p* = 0.08
Class 0–1	2	2	0	
Class 2	4	4	0	
Class 3 or 3 + 5	8	6	2	
Class 4 or 4 + 5	14	5	9	
Class 5	2	1	1	
All persistent proliferative LN				
*NIH activity (median, IQR)*	3.5 (2.0–5.0)	3.0 (2.0–4.0)	5.0 (3.3–8.0)	***p* = 0.01**
*Change in NIH activity score (median, IQR)*	− 4 (− 7.0– − 1.0)	− 4.0 (− 7– − 1.8)	− 3.0 (− 6.0–3.0)	*p* = 0.28
*NIH chronicity score (median, IQR)*	3.0 (1.8–5.0)	2.0 (1.0–3.3)	4.0 (2.3–5.8)	***p* = 0.03**
*Change NIH chronicity score (median, IQR)*	1.0 (0–3.0)	1.0 (−0.3–2.3)	2.0 (1.0–5.0)	***P* = 0.03**
*Crescents on second biopsy*				*p* = 0.052
Yes (*n*, %)	7 (23.3%)	2 (11.1%)	5 (41.7%)	
No (*n*, %)	23 (76.7%)	16 (88.9%)	7 (58.3%)	

Among the 12 patients included in the IRR group, five met criteria for no response, two met criteria for mild response, and five for moderate renal response.

At the time of second biopsy, both groups had lower SLEDAI scores compared to first biopsy, and the CRR group had significantly lower SLEDAI scores compared to the IRR group. The median SLEDAI score of the CRR group was 2 (IQR 0–4.0) compared to a median of 8 (IQR 6.0–12.0) in the IRR group (*p* < 0.01). The higher SLEDAI score in the IRR group was attributable to ongoing kidney manifestations of LN including proteinuria and pyuria.

Of the other laboratory parameters assessed at time of second biopsy, including age, dsDNA, serum C3, change in C3 between biopsies, and serum C4, none were found to be significantly different between the two groups.

There was no significant difference in maintenance therapy at time of second biopsy between the CRR and IRR groups.

### Kidney pathology on second biopsy

The most common pathologic finding on second biopsy was persistent proliferative glomerulonephritis in both groups. In the CRR group, 11/18 of the subjects had persistent proliferative disease on second biopsy. The remaining 7/18 showed resolution of proliferative findings. In the IRR group, 11/12 of subjects with proliferative nephritis on initial biopsy had persistent proliferative nephritis on the follow-up biopsy (Table 2). Only one patient in the IRR group had improvement to isolated class V on second biopsy. No patients in either remission group developed new proliferative nephritis on second biopsy.

Both groups showed improved NIH activity scores compared to baseline at the time of second biopsy; however, the CRR group had significantly lower activity scores than the IRR group.

Median activity score on second biopsy in the CRR group was 3 (IQR 2.0–4.0) while in the IRR group it was 5.0 (IQR 3.3–8.0) (*p* = 0.01). Both groups had worse NIH chronicity scores on second biopsy, again with the CRR group faring significantly better. The median chronicity score on second kidney biopsy in the CRR group was 2 (IQR 1.0–3.3) while in the IRR group median chronicity score was 4 (IQR 2.3–5.8) (*p* = 0.03). The CRR group had less progression of chronicity on biopsy, with a change of 1.0 (− 0.3–2.3) compared to change in the IRR group of 2.0 (1.0–5.0) (*p* = 0.03).

There was a non-significant trend toward lower frequency of crescents visualized on second biopsy in the CRR group (2/16) than the IRR group (7/12) (*p* = 0.052).

The remaining histologic parameters on second biopsy, including change in the NIH activity score between first and second biopsy, median IgG, IgA, IgM, C3, and C1q staining showed no significant differences between the CRR and IRR groups.

### Complications of kidney biopsy

Of the 60 kidney biopsies analyzed, there were no serious complications requiring hospital admission or other intervention.

### Association of laboratory parameters with persistent proliferative disease

Given the limited performance of CARRA response in predicting which patients have proliferative disease on second biopsy, the relationship between other laboratory measures and histologic lupus activity were examined. Using single predictor logistic regression models, only the SLEDAI score at time of second biopsy was found to be associated with persistent proliferative nephritis on second biopsy, OR 1.39 (CI 95 1.08–2.02) (Table 3). No other laboratory parameters, including individual components of SLEDAI score related to kidney function, were found to be significantly associated with persistent proliferative nephritis. Analysis included C3 (*p* = 0.74), C4 (*p* = 0.26), dsDNA (*p* = 0.48), creatinine (*p* = 0.65), uPCR (*p* = 0.49), urine red blood cell count (*p* = 0.07), and urine white blood cell count (*p* = 0.51) at the time of second biopsy. Similarly, the change in these measures between first and second biopsy was not significantly associated with proliferative nephritis on second biopsy.

**Table 3:** Association between laboratory parameters of lupus activity and persistent proliferative nephritis on second biopsy

Component	OR (CI 95)	*p-*value
C3	1.01 (0.97–1.04)	0.74
Delta C3	0.99 (0.97–1.02)	0.73
C4	1.05 (0.97–1.17	0.26
Delta C4	1.03 (0.96–1.12)	0.45
DsDNA	1.00 (0.99–1.01)	0.48
Delta dsDNA	0.99 (0.99–1.01	0.52
sCreat	2.7 (0.04 – 31.18)	0.65
Delta sCreat	0.13 (0.01–1.72)	0.21
UPCR	1.1 (0.89–1.69)	0.49
Delta UPCR	0.96 (0.84–1.09)	0.52
SLEDAI	**1.39 (1.08–2.02)**	**0.03**
Delta SLEDA	0.97 (0.87–1.06)	0.49
Urinary RBCs	1.13 (0.97–1.32)	0.07
Urinary WBCs	1.02 (0.98–1.09)	0.51
Microalbumin	0.99 (0.99–1.01)	0.15

No significant association was found between laboratory measures of lupus activity (C3, C4, dsDNA, uPCR, serum creatinine, and SLEDAI score) and persistent proliferative nephritis on second biopsy, using a multiple predictor logistic regression model.

### Impact of second biopsy on treatment

**Fig. 1:**
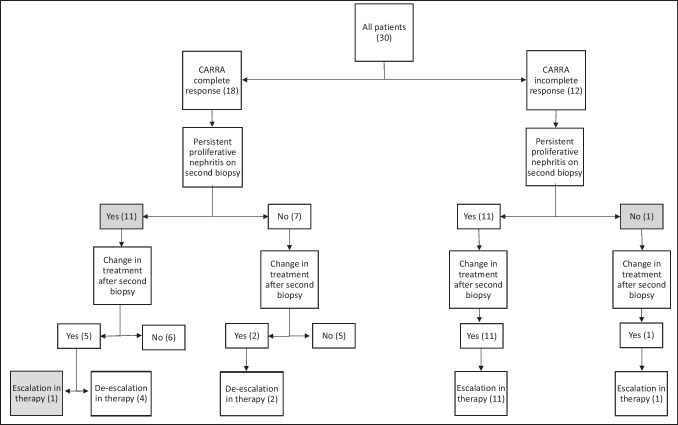
Second kidney biopsy results and impact on treatment decisions in CARRA complete and incomplete response groups with and without persistent proliferative lupus nephritis on kidney biopsy. Shaded boxes represent findings or decisions that were discordant with expectations based on clinical response status

Findings on second biopsy influenced immunosuppressive therapy decisions in both CRR and IRR groups (Fig. 1). Among the 11 CRR patients with persistent proliferative disease, 1/11 had an escalation in therapy (belimumab initiated), 4/11 had therapy de-escalated (three decreased or stopped prednisone, one decreased mycophenolate dose), and 6/11 had no change in treatment. Those with escalation and no change in therapy (7/18) would have otherwise been considered for de-escalation in therapy based on their laboratory parameters. In the CRR with a resolved nephritis group, 2/7 had a de-escalation in their therapy (stop prednisone) and 5/7 of patients had no change in therapy. Decrease in activity score was the most common factor that led to a de-escalation of therapy; although, not all patients with a lower activity score had therapy de-escalated. The one patient with CRR and an escalation in therapy had an increase in both activity score and chronicity score on second biopsy compared to first biopsy. Of the patients who had no change in treatment in this group, 10/11 had a decrease in activity score and 1/11 had an increase in activity score. Of these patients, 8/11 had an increase in chronicity score while 3/11 had no change in chronicity score. Changes in chronicity score in this group varied, with 2/6 having an increase in chronicity score, 3/6 having a decrease in chronicity score, and 1/6 having no change in chronicity score.

All 12 patients in the IRR group had an escalation in treatment based on the results of the second biopsy. Treatment escalations included increased maintenance MMF dose, higher dose corticosteroid therapy (either repeat oral prednisone burst and taper and/or pulse methylprednisolone), initiation of rituximab, cyclophosphamide, or cyclosporine. Treatment decisions were based on prescribing physician preferences and patient’s past clinical history. The difference in treatment implications between the CRR and IRR groups after second biopsy was significant (*p* ≤ 0.01, *χ*^2^ = 26.15). Of those with available data, 3/11 had an increase in activity score between first to second biopsy and 7/11 had a decrease in activity score. All patients had an increase in chronicity score between first and second biopsy.

### Review of patient laboratory outcomes following second biopsy

CARRA renal response was evaluated at last known follow-up after the second biopsy. In the cohort of patients who had achieved CRR at time of second biopsy, 11/18 remained in complete renal response at median follow-up 4 years after kidney biopsy. Of the 11 CRR patients with persistent nephritis, one had escalation of therapy and moderate remission at last follow-up. Of the four with reduction in immunosuppression despite persistent proliferative LN, one had full renal relapse at follow-up, one met criteria for moderate remission, and two remained in full renal remission by CARRA definitions. Of the six with no change to therapy, five remained in complete remission and one with moderate remission at last follow-up. Among the seven patients with resolution of proliferative LN, four remained in complete remission at last follow-up, one had relapses to no renal response, and one had moderate renal remission.

Among patients in the IRR group at time of second biopsy, at last follow-up (mean 6.5 years), 3/12 were in complete renal remission (Supplemental Table 2).

No patients progressed to kidney failure between first and second biopsy. The patient in the IRR group who had resolution of proliferative nephritis and transition to class V LN developed kidney failure requiring dialysis 22 months after the second biopsy.

## Discussion

These data demonstrate that routine follow up kidney biopsy approximately 1 year after initial diagnosis of pediatric onset proliferative LN was most likely to show persistent proliferative glomerulonephritis, independent of CARRA renal response status. This was seen uniformly in subjects who had not achieved complete renal remission and was also a common finding in subjects in whom laboratory parameters indicated quiescent kidney disease. This finding is in keeping with other analyses in adults with LN that have demonstrated discordance between kidney biopsy findings at time of initial diagnosis [7] and on follow-up biopsy [6]. There were no significant laboratory predictors of ongoing proliferative nephritis found in this group. Among patients with and without complete remission, results of the second kidney biopsy influenced immunosuppressive treatment.

In adult cohorts, persistent nephritis on follow-up biopsy has been shown to predict LN flare in the setting of decreased immunosuppression [5] and decline in eGFR over time [14]. However, current clinical practice guidelines [15] recommend prescribing immunosuppression based on laboratory remission status alone, reserving repeat kidney biopsy for scenarios of refractory clinical nephritis or where an alternative diagnosis is suspected. This may lead to an underestimation of long-term risk of kidney disease and under immunosuppression, particularly in the patients with clinical remission after initial induction therapy.

On average, patients with complete renal remission demonstrated lower activity on biopsy; however, there were patients in the CRR group who had ongoing proliferative nephritis including elevated activity scores. In both remission status groups, glomerular antibody deposition remained present and was statistically similar in intensity. The implications of persistent immune deposits independent of clinical response highlights the difficulty in accurate understanding of immune activity based on laboratory parameters alone. This may indicate failure of initial induction immunosuppressive regimens and ongoing disease activity or may suggest the presence of antibodies in the glomeruli could reflect failure to clear antibody in the time interval between initial and second biopsy. While NIH disease activity and chronicity scores have been associated with long-term kidney outcomes, there are no data to indicate the relationship between presence of persistent glomerular deposits and clinical outcomes. Unsurprisingly, a long-term remission status was associated with CARRA remission status at 1 year after initial biopsy.

Most of the patients in this cohort had immunosuppressive therapy escalated after the second biopsy. The major potential benefit of follow-up kidney biopsy in this cohort was to identify the group of patients with clinically quiescent disease and persistent histologic activity. This group made up 11/30 of the total cohort. Without the biopsy information, this group may have had a reduction in immunosuppression dosing that could have put them at risk for a subsequent flare. However, four patients with persistent proliferative nephritis on second biopsy had therapy de-escalated despite the biopsy findings, suggesting that other factors influenced the physicians’ decisions regarding immunosuppression in this group. Among those who had not achieved renal remission, confirmation of ongoing proliferative nephritis on biopsy resulted in change to therapy, typically to an alternative first-line agent. Theoretically, a kidney biopsy that demonstrated advanced chronic changes might result in decisions to avoid escalating immunosuppression to avoid medication toxicity that might not be beneficial. In this group with sub-nephrotic range proteinuria and preserved kidney function, kidney biopsy confirmed the clinical suspicion based on laboratory findings and ultimately did not impact clinical decisions regarding immunosuppression, which likely would have been escalated without the biopsy. Strategies to use kidney biopsy after 3 years of therapy to guide tapering of maintenance immunosuppressive therapy have been proposed in adult patients [16]. Given the higher risk of progressive LN in children, additional investigations for the applicability of such protocols to the pediatric population will be necessary.

The rate of complete histologic remission (activity score of 0) in our cohort was lower than expected at 1/30, compared with previously reported rates of approximately 20% [6]. The reason for lower rates of complete histologic remission may be related to earlier timing of repeat kidney biopsy immediately after induction in the comparative group, subject age, medication adherence, and predominantly LatinX race in our patients, for whom genetic, environmental, and socioeconomic factors may influence their disease course and response to initial induction immunosuppression. The expected timing of resolution of kidney biopsy findings after therapy for LN is unknown. A recent report of serial biopsies in adult patients with LN demonstrated more rapid improvement in activity in response to initial treatment, with slower reduction in activity over the following 2–3 years, suggesting the timing of follow-up biopsy in our cohort may be relatively early in the histologic course of the disease [15]. The overall higher rates of persistent proliferative disease in our cohort may also reflect worse outcomes for LN in the pediatric population. In populations with lower rates of persistent proliferative nephritis on follow-up biopsy, the utility of follow-up biopsy for patients with clinical remission may be lower.

The failure to convert patients from IRR to CRR after initial biopsy may have been due to inadequate rescue treatment or may indicate that once the initial treatment response window has elapsed, a series of irreversible changes occur within the kidney so that no increase in immunosuppressive treatment achieves complete response. An alternative explanation for these outcomes is that patients who fail to achieve a complete response have more refractory underlying disease despite similar initial clinical and pathologic findings.

### Laboratory predictors of proliferative disease

We did not find laboratory parameters that predicted persistent proliferative nephritis on kidney biopsy apart from the SLEDAI score with a single predictor logistic regression. However, individual components of the SLEDAI score, including those reflective of kidney disease, did not predict persistent proliferative nephritis when analyzed in a multiple predictor model. This analysis is limited by the small number of patients analyzed. This suggests that more active systemic manifestations of SLE may be associated with persistent proliferative LN. Not surprisingly, the CARRA complete renal remission status was the strongest predictor of resolution of proliferative nephritis on follow-up biopsy; however, even this composite risk factor identified fewer than half of subjects with persistent proliferative nephritis. Other studies have reported that proteinuria [16] and lower serum complement [7] are associated with persistent nephritis; however, those associations were not found in our analysis. Because this was a retrospective review, we did not have access to other biomarkers that may be associated with persistent nephritis.

### Risks of follow-up biopsy

The primary risk of a repeat biopsy is bleeding complications. In our cohort of 30 patients with 60 native kidney biopsies, none had a serious biopsy complication defined as requiring hospital admission or other intervention. While this patient cohort did not experience complications, we acknowledge that kidney biopsy is not risk free. Our hospital-wide risk of complication requiring hospital admission during this time period was 1.7% and the reported risks of pediatric kidney biopsy complication requiring a blood transfusion or other intervention are 0.9% and 0.7% respectively (0.7%) [17]. If the information obtained on biopsy can impact patient treatment and outcomes, the risk of kidney biopsy is justified until non-invasive markers of persistent nephritis can be reliably identified.

### Limitations

There are several important limitations to this analysis including that it is a retrospective analysis of patients at a single center. Another major limitation of our analysis is the overall small size and low percentage of total population who met criteria for inclusion. Only 44 of the 135 patients with lupus nephritis at our institution underwent a second biopsy in the designated time frame and only 30 of these biopsies were performed within the 1 ± 0.3-year window. The narrow inclusion criteria may have excluded both low-risk patients (i.e., those with good initial response to therapy resulting in clinician decision to defer repeat biopsy) and high-risk patients (i.e., those with poor follow-up or those who required a second biopsy earlier than the 1 ± 0.3-year window). KDIGO recommends observing patients who are having some response to therapy but have not met criteria for complete remission. The study is underpowered for multivariate analysis.

Another major shortcoming in our ability to draw conclusions from these data is the absence of long-term patient and kidney outcomes from this analysis. Most patients remained in their clinical remission classification during the 3-year period following second kidney biopsy. The lack of clinically evident kidney relapse in the CRR group may be related to targeted adjustments in kidney biopsy as opposed to reliance on laboratory parameters. Outcomes beyond three years in this group are not known.

Finally, the retrospective nature of the study is another limitation as all data was collected via chart review. Ideally, future research in this area would be in the form of prospective studies.

### Further questions

The optimal timing and utility of follow-up kidney biopsy to monitor disease activity and tailor treatment in LN is unknown. Ideally, kidney biopsy would be timed to coincide with critical junctures in clinical decision-making so that the risks associated with escalating, changing, and/or reducing immunosuppressive therapy could be informed by a complete perspective on disease activity. In addition to defining the right moment in the disease for biopsy, a better understanding of the clinical implications of the pathologic characteristics on biopsy throughout the disease course will be critical to tailor therapy to individual patients. Ideally, the development of reliable non-invasive clinically available biomarkers of disease activity could be critical to replace repeated invasive procedures and guide decisions regarding more precisely targeted therapies.

Given the worse outcomes for children with LN compared to adults, we suggest additional larger and long-term pediatric-specific studies to define the role of repeated kidney biopsy in children with LN. As more therapies become available for treatment of LN, determining when and how to use them to treat children who may require a lifetime of immunosuppression will be critical.

## Conclusion

Currently available laboratory parameters of disease remission in LN after induction therapy are insufficient to identify children with ongoing evidence of persistent proliferative nephritis. Follow-up kidney biopsy can be used to guide therapy in children with SLE and nephritis.

## Supplementary information

Below is the link to the electronic supplementary material.

Graphical abstract (PPTX 88 KB)

Supplementary file2 (DOCX 47 KB)
