# Supplementary material for: Incomplete concordance between laboratory and pathologic findings on post-induction kidney biopsy in pediatric patients with proliferative lupus nephritis
Source: Pediatr Nephrol. 2025 Mar 25;40(9):2845–54. doi: 10.1007/s00467-025-06736-y (PMC12296845; doi:10.1007/s00467-025-06736-y)
Supplement: Supplementary file 1 — Graphical abstract (PPTX 88 KB) [file 467_2025_6736_MOESM1_ESM.pptx]

## Slide 1
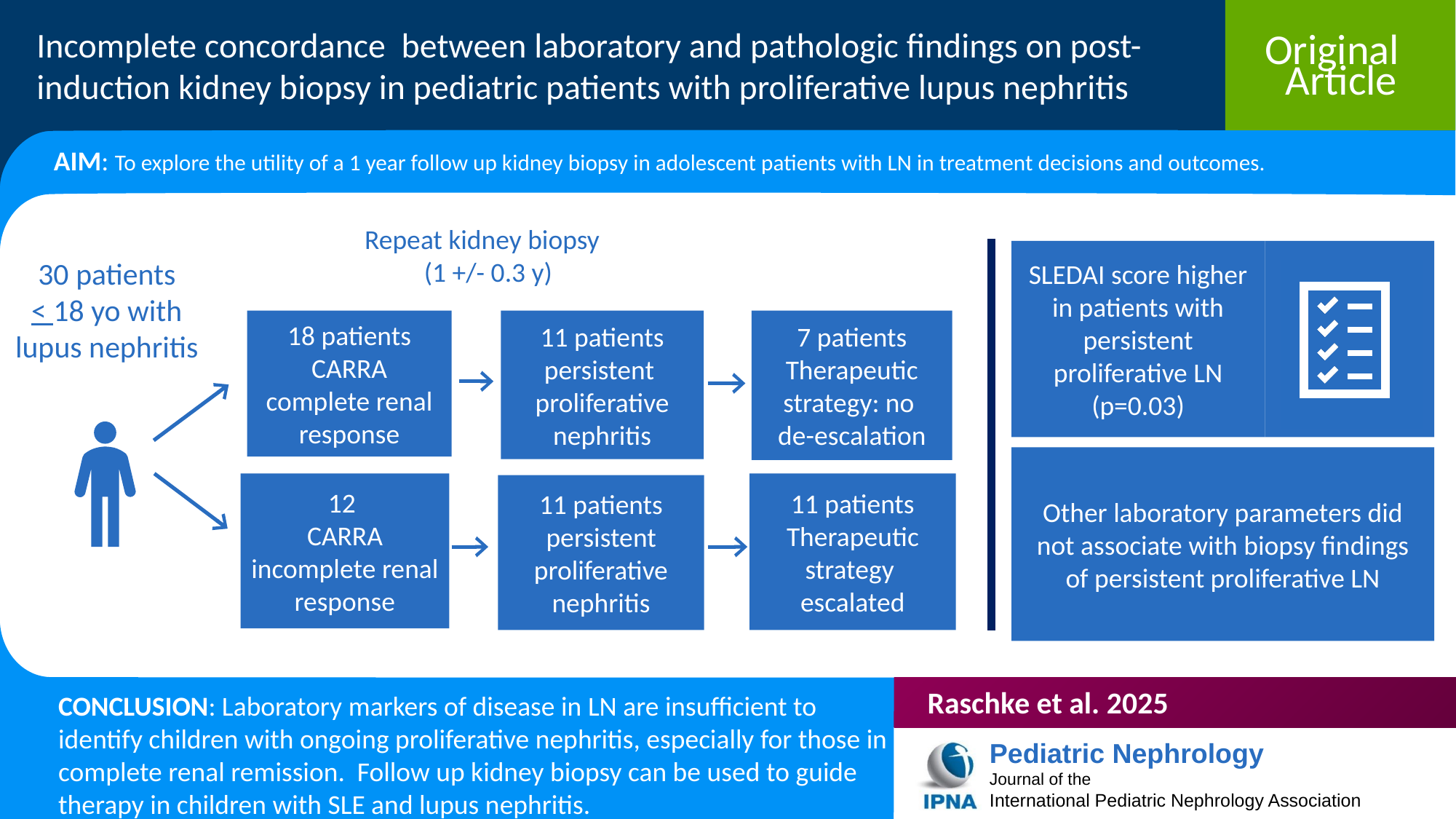

Incomplete concordance between laboratory and pathologic findings on post-induction kidney biopsy in pediatric patients with proliferative lupus nephritis
AIM: To explore the utility of a 1 year follow up kidney biopsy in adolescent patients with LN in treatment decisions and outcomes.
Repeat kidney biopsy
(1 +/- 0.3 y)
SLEDAI score higher in patients with persistent proliferative LN (p=0.03)
30 patients
< 18 yo with lupus nephritis
18 patients
CARRA complete renal response
11 patients
persistent proliferative nephritis
7 patients
Therapeutic strategy: no
de-escalation
Other laboratory parameters did not associate with biopsy findings of persistent proliferative LN
12
CARRA incomplete renal response
11 patients
Therapeutic strategy escalated
11 patients
persistent proliferative nephritis
Raschke et al. 2025
CONCLUSION: Laboratory markers of disease in LN are insufficient to identify children with ongoing proliferative nephritis, especially for those in complete renal remission. Follow up kidney biopsy can be used to guide therapy in children with SLE and lupus nephritis.
