# Supplementary material for: Incomplete concordance between laboratory and pathologic findings on post-induction kidney biopsy in pediatric patients with proliferative lupus nephritis
Source: Pediatr Nephrol. 2025 Mar 25;40(9):2845–54. doi: 10.1007/s00467-025-06736-y (PMC12296845; doi:10.1007/s00467-025-06736-y)
Supplement: Supplementary file 2 — Supplementary file2 (DOCX 47 KB) [file 467_2025_6736_MOESM2_ESM.docx]

Supplemental Table 1.  Immunofluorescence Ab staining at initial and follow up kidney biopsy for lupus nephritis in CARRA complete response and incomplete response groups. *(p<0.05)

|  | **All Patients** | |  | **CARRA Response: Complete** | |  | **CARRA Response: Incomplete** | |
| --- | --- | --- | --- | --- | --- | --- | --- | --- |
| **Immunofluorescence Ab**  **Median (IQR)** | 1^st^ Biopsy | 2^nd^ Biopsy |  | 1^st^ Biopsy | 2^nd^ Biopsy |  | 1^st^ Biopsy | 2^nd^ Biopsy |
| IgG | 2.5 (1.0, 2.0) | 2.0 (1.0, 2.0) |  | 3.0 (1.0, 3.1)* | 2.0 (1.0, 2.0) |  | 2.0 (2.0, 2.5)* | 1.8 (1.0, 2.0) |
| IgM | 2.0 (1.0, 2.5) | 1.0 (0.5, 2.0) |  | 2.0 (1.4, 3.0) | 1.0 (0.5, 1.6) |  | 2.0 (0.6, 2.5) | 1.0 (0.5, 2.0) |
| IgA | 2.3 (1.5, 3.0) | 1.0 (0.5, 2.0) |  | 2.8 (1.4, 3.0) | 1.0 (0, 1.6) |  | 2.0 (1.5, 2.5) | 1.8 (0.6, 2.0) |
| C3 | 3.0 (2.0, 3.0) | 1.3 (0.5, 2.0) |  | 3.0 (2.0, 3.1) | 1.5 (0.9, 2.0) |  | 2.3 (1.6, 3.0) | 0.8 (0.1, 2.4) |
| C1q | 2.3 (2.0, 3.0) | 1.0 (0, 2.0) |  | 2.5 (2.0, 3.0) | 1.0 (0, 2.0) |  | 2.0 (1.0, 2.9) | 1.5 (0.1, 2.0) |

Supplemental Table 2. CARRA status at last follow up by CARRA response category at time of second kidney biopsy.

|  |  | CARRA Response: Complete (n=18) |  | CARRA Response: Incomplete (n=12) |  | P value |
| --- | --- | --- | --- | --- | --- | --- |
| Age(years) at Last Follow-Up, Median (IQR) |  | 19.4 (16.8, 20.6) |  | 20.8(20.25, 23.75) |  |  |
| Time (years) since 2^nd^ Kidney Biopsy, Median (IQR) |  | 4.0 (2.2, 5.5) |  | 6.5(2.9, 8.6) |  |  |
| Patients (n) with CARRA complete response by:  eGFR ≥90 mL/min/1.73 m^2^  UPCR <0.2  Hematuria <5 RBC/HPF  Pyuria <5 WBC/HPF |  | 16  15  17  15 |  | 8  5  8  8 |  | P=0.184  **P=0.045***  P=0.128  P=0.392 |
| Patients Last CARRA Response Status (n)  Complete  Moderate  Mild  None |  | 11  5  0  2 |  | 3  4  1  4 |  | P<0.001* |
